# Topical Steroids and Antibiotics for Adult Blepharokeratoconjunctivitis (BKC): A Meta-Analysis of Randomized Clinical Trials

**DOI:** 10.1155/2021/3467620

**Published:** 2021-01-08

**Authors:** Lu Zhao, Ya-jie Sun, Zhi-qiang Pan

**Affiliations:** ^1^Beijing Tongren Eye Center, Beijing Key Laboratory of Ophthalmology and Visual Sciences, Beijing Tongren Hospital, Capital Medical University, Beijing 100730, China; ^2^Department of Ophthalmology, Henan Provincial People's Hospital, Henan Eye Institute, People's Hospital of Zhengzhou University, Zhengzhou, China

## Abstract

**Purpose:**

A meta-analysis was conducted to evaluate the efficacy and safety of topical treatments (including steroids and antibiotics) for adults with blepharokeratoconjunctivitis (BKC).

**Methods:**

The following databases were searched for relevant randomised controlled trials (RCTs): China National Knowledge Infrastructure (CNKI), Web of Science, MEDLINE, PubMed, Embase, and Cochrane Central Register of Controlled Trials database (CENTRAL). Two reviewers selected studies and analyzed the risk of bias independently. The treatments were loteprednol 0.5%/tobramycin 0.3% (LE/T) and dexamethasone 0.1%/tobramycin 0.3% (DM/T). The efficacy outcome measures were change from baseline (CFB) in composite scores of ocular symptoms and signs; the CFB in the signs composite scores for blepharitis, conjunctivitis, and keratitis at each visit; the total ocular adverse event incidence (AEs); and the incidence of intraocular pressure (IOP) increase after treatment. Prepost mean differences (MDs) were compared for continuous outcome variables, and incidences were analyzed for dichotomous data. The pooled effect sizes were analyzed using 95% confidence intervals (CIs) in a fixed-effect model. Heterogeneity was evaluated using the *Q*-test and *I*^2^ statistic.

**Results:**

The CFB to final visit in ocular symptoms and signs of BKC was not statistically different between the two treatments (95% CI, −0.33 to 1.50; MD = 0.58; *P*=0.21). The CFB in signs composite scores for blepharitis (95% CI, −0.16 to 0.48; MD = 0.16; *P*=0.32), conjunctivitis (95% CI, −0.55 to 1.76; MD = 0.61; *P*=0.30), and keratitis (95% CI, 0.00–0.28; MD = 0.14; *P*=0.05) was also similar with the two treatments. LE/T was a safer intervention than DM/T, with fewer overall adverse events (95% CI, 0.34–0.80; RR = 0.52; *P*=0.003) and significantly less elevation of intraocular pressure (IOP) (95% CI, 0.32–0.70; RR = 0.47; *P*=0.0002).

**Conclusions:**

DM/T and LE/T are both effective treatments for BKC, but LE/T may be a safer intervention.

## 1. Introduction

Blepharokeratoconjunctivitis (BKC) is an inflammatory disorder of the eyelid margin with secondary conjunctivitis and keratopathy [1]. The main clinical manifestations include an inflamed palpebral margin, conjunctival congestion, conjunctival follicles, punctate corneal epithelial erosion, corneal stromal infiltration, corneal ulcer, and eventually scarring with consequent loss of vision and possibly blindness [2–4]. The severity of BKC can be categorized as mild, moderate, and severe, according to the depth and extent of keratopathy [5]. Inappropriate therapy or delayed diagnosis may cause serious corneal disorders [4].

Conventional treatments for BKC include nonsteroidal anti-inflammatory drugs (NSAIDs), antibiotics, and corticosteroids and are often prescribed in combination with artificial eye drops and eyelid hygiene, massage, or light therapy [6, 7]. Several clinical trials have been performed to evaluate the safety and efficacy of topical drugs in the treatment of BKC in adults. No consensus, however, has been reached. Pranoprofen can significantly relieve ocular surface inflammation and improve the cure rate of BKC [8]. 1% fluorometholone eye drops combined with levofloxacin eye drops were also effective [9]. Topical antibiotics eradicate positive cultures of bacteria from the palpebral margins, and different types of antibiotics (such as ciprofloxacin or tobramycin) have similar impact on the number of bacteria.

Topical steroids have also proven useful in relieving ocular symptoms, but ineffective in eliminating harmful bacteria from eyelids [10]. Clinically, patients with mild BKC are generally treated with nonsteroidal anti-inflammatory drugs, which are safer than glucocorticoids [8]. However, for moderate to severe BKC, glucocorticoids are generally used for their effectiveness as anti-inflammatory agents [8]. The present systematic review and meta-analysis was undertaken to evaluate the efficacy and safety of topical treatments for BKC. The evidence reviewed was from published RCTs on the effectiveness of topical treatments for adult BKC.

## 2. Materials and Research Methods

### 2.1. Search Strategies

The following databases were searched for RCTs relevant to the review question: PubMed, China National Knowledge Infrastructure (CNKI), Web of Science, Embase, MEDLINE, and CENTRAL. The primary search strategy in PubMed used the relevant medical subject heading (MeSH) terms “blepharokeratoconjunctivitis” and “therapeutics.” The detailed electronic search strategy in PubMed was as follows including Boolean operators: (blepharokeratoconjunctivitis[Title]) OR Blepharitis-Associated Keratoconjunctivitis[Title]) OR BKC[Title]) OR blepharokeratitis[Title]) OR blepharoconjunctivitis[Title]) AND (Therapeutics[Title/Abstract]) OR Therapies[Title/Abstract]) OR Treatments[Title/Abstract]) OR Therapeutic[Title/Abstract]) OR Therapy[Title/Abstract]) OR Treatment[Title/Abstract]). The reference lists of selected articles were also checked. The search was conducted with no restrictions on dates and languages. The United States National Institutes of Health register of clinical trials (ClinicalTrials.gov) was also searched to look for any ongoing or possibly unpublished relevant trials. The PubMed, MEDLINE, CNKI, Web of Science, Embase, and CENTRAL search strategies are presented in Supplementary Materials.

### 2.2. Study Selection

Two reviewers (LZ and YJS) selected studies independently using predetermined selection criteria (see Section 2.3). The titles and abstracts were screened to assess relevance and eligibility, and the full article was accessed if necessary to check eligibility. If the two reviewers did not agree, discussion with an additional reviewer (ZQP) allowed a final decision to be reached.

### 2.3. Inclusion and Exclusion Criteria

Studies meeting the following criteria were included: (1) the study included patients who were at least 18 years old; (2) patients had a diagnosis of BKC prior to the treatments; (3) the presence of ocular signs was evidenced by slit-lamp examination; (4) patients had received topical ocular treatments; and (5) the study design was RCT.

Trials were excluded if:

1. The patients had previously or concurrently taken oral or topical treatments
2. The data could not be included in statistical analysis due to incomplete reporting (e.g., lack of standard deviation).

### 2.4. Data Extraction

Two reviewers (LZ and YJS) selected the studies based on the criteria outlined in Section 2.3. Each reviewer then extracted variables independently using a previously designed data extraction tool. The extracted variables included sample size, locations of studies, patients' mean age and gender balance, types of intervention, drug dosage, duration of follow-up, primary and secondary outcomes, and assessment methods. If these variables were not reported, the reviewers contacted the trial authors via email to request the data.

### 2.5. Assessment of Risk

Risk of bias was accessed by two reviewers (LZ and YJS) independently based on the Cochrane Handbook criteria focusing on seven aspects as follows: allocation concealment, blinding of outcome assessors, incomplete outcome data, random sequence generation, participant and personnel blinding, selective reporting, and other sources of bias. Each included trial was critically appraised by the reviewers considering each of the above areas. Any unresolved disagreement between the two reviewers was resolved following review by author ZQP.

### 2.6. Statistical Analyses

The primary outcome measure was CFB of the ocular symptoms and signs composite score at the final follow-up visit (day 15 ± 1). The secondary outcome measures were the CFB in composite score of ocular signs of blepharitis, conjunctivitis, and keratitis at each follow-up. The safety outcome measure was overall incidence of ocular AEs (including increased IOP). The weighted mean difference (WMD) of continuous data with 95% CI was determined using the inverse variance method. For dichotomous data, the risk ratio (RR) was calculated with 95% CI and the weighted summary RR was determined using the Mantel–Haenszel method. The *Q-*test and *I*^2^ statistic were used to assess heterogeneity, with values of 25%, 50%, and 75% indicating low, medium, and high levels, respectively. At *I*^2^ level greater than 50%, meta-analysis was conducted using the fixed-effects model, and at lower levels, the random-effects model. Statistical analyses in this study were conducted using Review Manager Software (RevMan 5.3; Cochrane Collaboration, Oxford, UK), and *P* values lower than 0.05 were considered significant.

## 3. Results

### 3.1. Search Results and Study Inclusion

**Figure 1:**
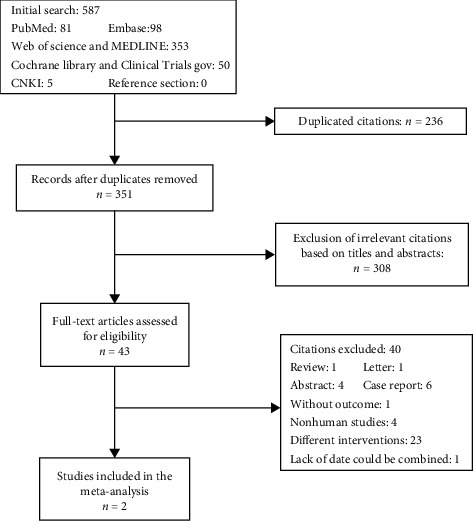
Flowchart of study inclusion.

The data searches yielded 587 studies (Figure 1) of which 236 duplicated citations were excluded. After the exclusion of irrelevant citations based on titles and abstracts, 43 articles remained. On full-text checking, 41 of these were excluded due to their type of intervention (23 articles), nonhuman studies (4 articles), lack of reported outcome (1 article), review (1 article), letter (1 article), abstract (4 articles), case report (6 articles), or unqualified outcomes (1 article). The remaining 2 studies were included in the meta-analysis (Figure 1).

### 3.2. Study Characteristics

Characteristics of the included studies are summarized in Table 1. Both were published in English [11, 12], one was conducted in China and the other in the U.S.A. In total, the studies involved 627 patients with BKC. The two studies used the same criteria for diagnosis of BKC and compared LE/T and DM/T ophthalmic solutions applied 4 times daily.

### 3.3. Risk of Bias

**Figure 2:**
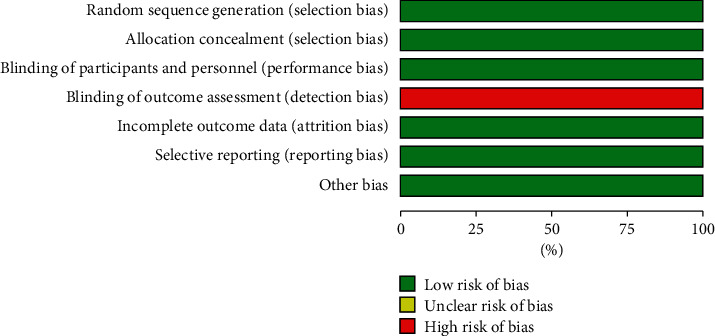
Graph illustrating risk of bias.

**Figure 3:**
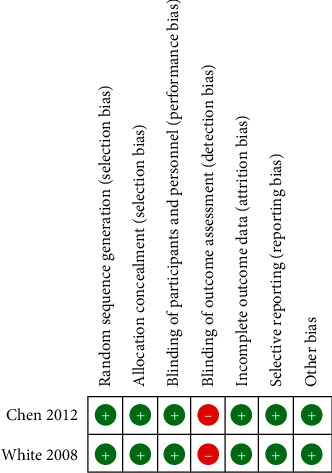
Summary of risk of bias.

Overall, risk of bias assessment found that the included studies were at low risk, and therefore, high quality (Figure 2), although outcome, assessments were not blinded (Figures 2 and 3).

### 3.4. Effect Outcomes

**Figure 4:**
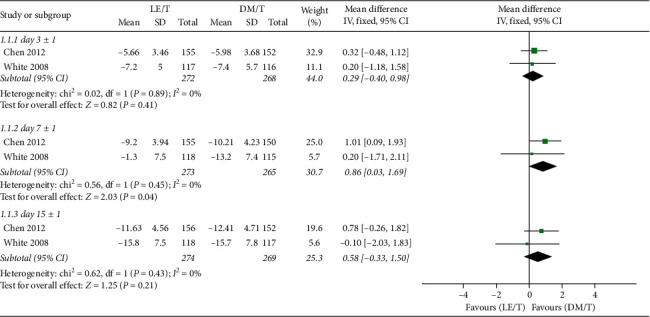
Forest plot of CFB in the ocular signs and symptoms composite score.

The primary outcomes of the meta-analysis are presented in Figure 4. Statistical heterogeneity (*I*^2^) was zero for most outcome measures, and a fixed-effects model was used for these. The RCTs tested effects of the two interventions by comparing the ocular signs and symptoms composite score between the start point and endpoints. The endpoints included the score at the second visit (day 3 ± 1), the third visit (day 7 ± 1), and the fourth visit (day 15 ± 1).

The CFB in ocular symptoms and signs composite scores was not significantly different between the LE/T and DM/T groups at the second (95% CI: −0.40–0.98, *I*^2^ = 0%; MD = 0.29) or the fourth follow-up visits (95% CI: −0.33 to 1.50, I^2^ = 0%; MD = 0.58). However, the LE/T group showed a higher improvement from baseline than the DM/T group at the third visit (95% CI: 0.03–1.69, I^2^ = 0%; MD = 0.86) (Figure 4).

**Figure 5:**
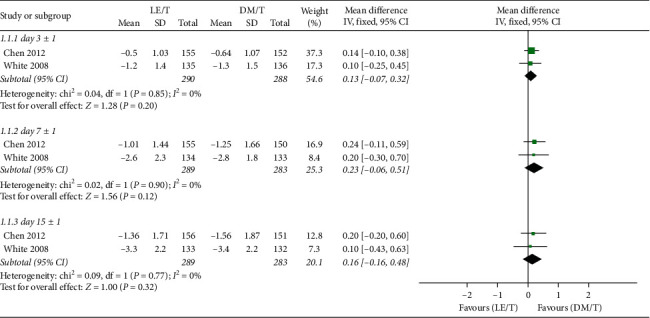
Forest plot of CFB in the blepharitis signs composite score.

CFB in the blepharitis signs composite scores was not significantly different between the LE/T and DM/T groups at the second (95% CI: −0.07 to 0.32, I^2^ = 0%, MD = 0.13), the third (95% CI: −0.06 to 0.51, I^2^ = 0%, MD = 0.23), or the fourth follow-up visit (95% CI: −-0.16 to 0.48, I^2^ = 0%, MD = 0.16) (Figure 5).

**Figure 6:**
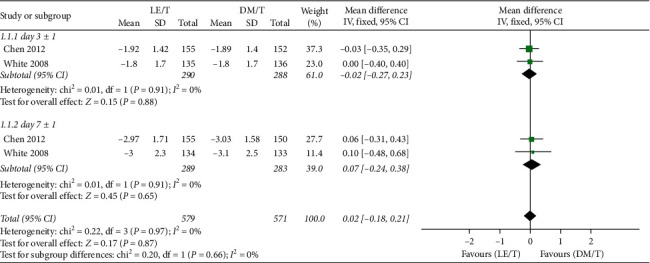
Forest plot of CFB in conjunctivitis signs composite score.

No significant difference in CFB of conjunctivitis signs was found between the two groups at the second visit (95% CI: −0.27 to 0.23, I^2^ = 0%, MD = −0.02), the third visit (95% CI: −0.24 to 0.38, I^2^ = 0%, MD = 0.07), or the fourth visit (95% CI: −0.55 to 1.76, I^2^ = 91%, MD = 0.61) (Figure 6).

**Figure 7:**
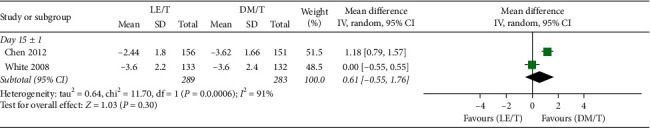
Forest plot of CFB in conjunctivitis signs composite score at day 15 ± 1.

Statistically significant heterogeneity was found in the conjunctivitis composite score at the final visit (day 15 ± 1), so a random-effect model was used (Figure 7).

**Figure 8:**
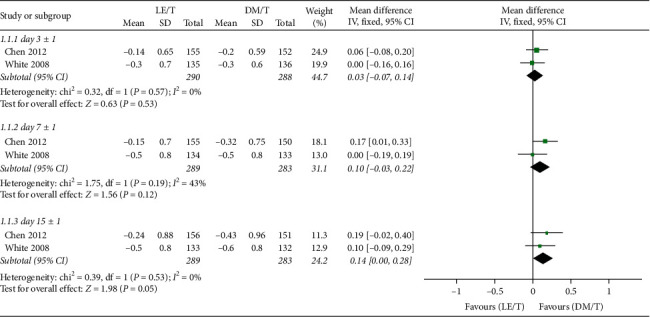
Forest plot of CFB in keratitis signs composite score.

No statistically significant difference in CFB between treatment groups was found in keratitis signs composite score at the second (95% CI: −0.07 to 0.14, I^2^ = 0%, MD = 0.03), third (95% CI: −0.03 to 0.22, I^2^ = 0%, MD = 0.10), or fourth visits (95% CI: 0.00–0.28, I^2^ = 0%, MD = 0.14) (Figure 8).

### 3.5. Safety Outcomes

**Figure 9:**
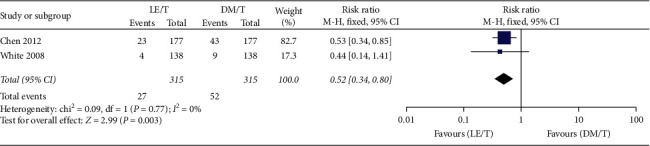
Incidence of overall adverse events in the groups treated with LE/T or DM/T.

**Figure 10:**
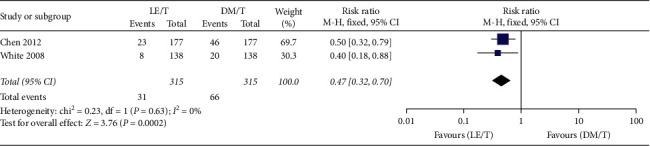
Incidence of elevating intraocular pressure (by 10 mmHg or less).

**Figure 11:**
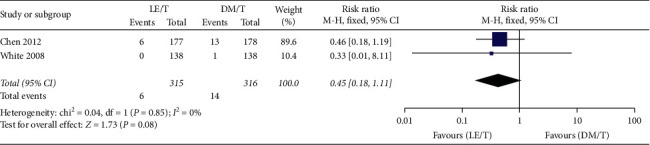
The incidence of elevating intraocular pressure (by more than 10 mmHg).

Adverse events were reported by both RCTs. The pooled data collected from 627 patients showed that overall adverse events happening in the LE/T treatment group (*n* = 27, 8.6%) were fewer than those in the DM/T group (*n* = 52, 16.5%), with 0.52 risk ratio (95% CI: 0.34–0.80, *P*=0.003) (Figure 9). The pooled analysis showed very low heterogeneity (*I*^2^ = 0, *P*=0.53). More patients treated with DM/T experienced a small (10 mmHg or less) increase in mean IOP than those treated with LE/T (RR = 0.47, 95% CI: 0.32–0.70, *P*=0.0002) (Figure 10). Similar numbers in the two groups suffered an elevation over 10 mmHg (RR = 0.45, 95% CI: 0.18–1.11, *P*=0.08) (Figure 11).

## 4. Discussion

BKC is an ocular disease that presents challenges to ophthalmologists in clinical practice. As its pathogenesis is not completely understood, clinical guidelines have not yet been provided. The purpose of BKC treatment is to modify meibomian gland function and the bacterial flora of the eyelid and conjunctiva and to reduce ocular inflammation [13]. Fortunately, with extensive research in recent years, knowledge about the disease is increasing. Yactayo-Miranda et al. [14] reported a higher rate of positive thioglycolate broth cultures in eyes with chronic blepharoconjunctivitis than in eyes without the disease. Treatment with 0.5% levofloxacin reduced the rate of positive thioglycolate broth cultures as well as the number of bacteria harbored on the conjunctiva. Azithromycin 1% ophthalmic solution has also been evaluated as a treatment for blepharitis or blepharoconjunctivitis and has been found to significantly decrease the number of coagulase-negative staphylococci and coryneform bacteria and improve the symptoms and signs of blepharitis [15–17]. Compared with this intervention, however, a combination of 0.3% tobramycin and 0.05% dexamethasone allows faster and more effective reduction of inflammation in moderate to severe blepharitis/blepharoconjunctivitis [17]. Another improvement on azithromycin 1.0% has been demonstrated by combining it with dexamethasone 0.1% resulting in more effective relief of clinical symptoms and eradication of bacteria [18]. In addition, an antibiotic-steroid combination of 0.1% dexamethasone plus 0.5% moxifloxacin is effective in reducing the inflammation associated with bacterial blepharitis [19]. Thus, a range of treatment methods exist, with different outcomes and levels of effectiveness in the treatment of BKS.

Existing systematic reviews on the safety and efﬁcacy of topical and systematic treatments for BKC in children found no high-quality evidence [6, 20]. Despite this, lubricants, antibiotics, and steroids have been considered as possible treatments for children and are available as eye drops or ointments [6]. The present meta-analysis included two articles involving the same intervention (antibiotics plus steroids) as well as the same outcome measures [11, 12]. A third study testing this intervention was not selected for inclusion in the meta-analysis, due to a lack of standard deviation data [21].

The present analysis reveals no significant differences between LE/T and DM/T as treatments of BKC in terms of ocular signs and symptoms. These results are inconsistent with those reported by Rhee and Mah [21] in which scores related to signs of blepharitis, conjunctivitis, and ocular discharge were reduced to a greater extent following DM/T treatment than LE/T treatment [21]. No AEs were reported, and the mean IOP change from baseline was similar in the two treatment groups [21]. The trial was a single-center study which included only 40 subjects with moderate or severe BKC, and the mean follow-up period was 3.5 days. In addition, the treatments were administered twice daily as opposed to four times per day in the two included trials. These differences in methodology may explain, at least in part, the differences between findings.

Incidences of nonocular treatment-emergent adverse events were comparable between treatments (8/315 in LE/T; 7/315 in DM/T) in the current meta-analysis. The nonocular side-effects were mostly mild to moderate. Only one hypertension subject in the LE/T group and one with a headache in the DM/T group were considered severe. Fewer ocular adverse events occurred in the LE/T group (6/315) than in the DM/T group (14/316). Most ocular AEs were mild to moderate, but IOP was increased, with significantly higher frequency in the DM/T than LE/T group. In addition, changes in IOP found among Chinese patients were higher than those among Americans, according to these two RCTs, in agreement with previous findings [22]. However, any possible association between race and elevation of steroid-related IOP has not yet been reported [23], but risk factors related to glaucoma, such as higher myopia, higher IOP and larger cup to disc area ratio are reportedly more common among Asians [24].

Corticosteroids may lead to multiple physiological changes involving the deposition of extracellular matrix material, the production of cross-linked actin fibers, inhibition of cell phagocytosis, and possibly to increased resistance to aqueous outflow and an elevation of IOP [25, 26]. Due to the different molecular structures of dexamethasone and loteprenol etabonate, the latter is associated with relatively low IOP elevation, making it a safer treatment for ocular inflammation, especially for the patients with a higher risk of IOP [12, 27].

Some studies have shown that topical calcineurin inhibitors (FK506, cyclosporin A 1%, pimecrolimus) have positive impacts on chronic blepharokeratoconjunctivitis treatments including longer remission periods than topical corticosteroids [28, 29], but this advantage needs to be supported by more studies with large samples.

Blepharitis is a common condition, and its treatment is an important clinical issue [2]. For Demodex-infested patients, eyelid scrubbing with a mild shampoo is not effective in reducing the number of mites, while many studies have shown that scrubbing the eyelid margin with tea tree oil can achieve this and improve ocular discomfort [30, 31]. Intense pulse light (IPL) is another effective and safe physical treatment for moderate to severe BKC adult patients and may be more useful in lessening eyelid margin inflammation than Meibomian gland expression [7].

Finally, dry eye secondary to BKC should also be considered. Artificial eye drops are an important treatment option because of the evaporative drying that accompanies meibomian gland disfunction and changes of the ocular surface microenvironment [32].

This review has some limitations. First, the number of clinical trials included is small. Only a few studies have focused on topical treatment of adult BKC. Second, this meta-analysis lacks subgroup analysis on different severities of BKC. Further high-quality randomised controlled trials with large stratified samples including different severity levels are warranted to determine the efficacy and limitations of the antibiotic plus steroid and other treatments for these different clinical categories of BKC.

## 5. Conclusion

This meta-analysis evaluated the evidence on efficacy and safety of topical antibiotics and steroids for adults with BKC. The results showed that LE/T and DM/T are similarly effective in the treatment of BKC, and that LE/T is associated with better safety.

## Figures and Tables

**Table 1:** Characteristics of the included studies.

First author	Publication year	Study design	Intervention		Regime duration	Other medication	Patient/eyes, *n*	Male/female, *n*		Mean age, years	
			LE/T	DM/T				LE/T	DM/T	LE/T	DM/T
Chen	2012	RCT	LE/T	DM/T	4 times/day 2 weeks	Limited	354/354	55/122	63/114	40.8 ± 13.6	41.7 ± 13.5
White	2008	RCT	LE/T	DM/T	4 times/day 2 weeks	Limited	273/273	53/83	52/85	55.3 ± 16.6	55.8 ± 16.5

DM/T: dexamethasone; 0.1%/tobramycin 0.3% LE/T: loteprednol etabonate 0.5%/tobramycin 0.3%.
